# Understanding Non-isothermal Crystallization Behaviors of Polyethylene-Derived Vitrimers

**DOI:** 10.1021/acs.macromol.5c01725

**Published:** 2025-08-20

**Authors:** Sara Valdez, Zhe Qiang

**Affiliations:** School of Polymer Science and Engineering, The University of Southern Mississippi, Hattiesburg, Mississippi 39406, United States

## Abstract

The transformation of polyolefins, including polyethylene (PE), into dynamic networks has become a promising research area in polymer sustainability due to their high availability, low cost, and desirable properties. Within these semicrystalline systems, the interplay between crosslinking and crystallinity becomes important as both factors can directly influence the thermal and mechanical performance of the materials. Furthermore, understanding the crystallization behaviors during processing is imperative, as varying cooling rates and conditions can influence the formation of crystalline regions. This study investigates the impacts of dynamic crosslinking and catalyst presence on the nonisothermal crystallization kinetics and mechanisms of PE-derived vitrimers and analyzes both the crosslinked and non-crosslinked components. All samples exhibited heterogeneous nucleation, and it was observed that crosslinking suppressed crystal growth rates, while the introduction of catalysts for transesterification can act as nucleating agents that influence the crystallization activation energy at various cooling rates.

## Graphical abstract

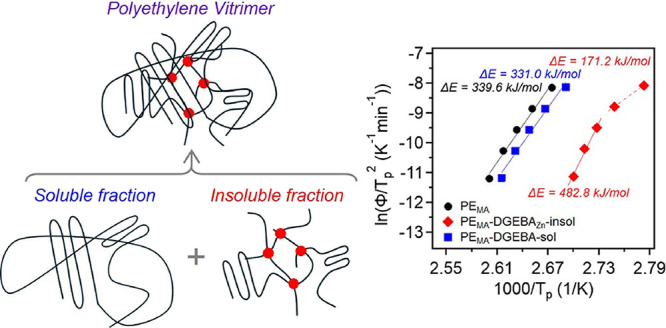

## Introduction

Polyolefins (POs) are some of the most widely used materials in the world with a wide range of applications,^1^ including packaging, textiles, rubbers, and automotive, making them ubiquitous in our everyday lives.^2^ Specifically, polyethylene (PE) and polypropylene (PP) dominate global markets due to their low production costs as well as satisfactory mechanical properties and excellent chemical resistance, which are largely attributed to their semicrystalline microstructure.^3,4^ Understanding and controlling the crystallinity of POs is a critical research area, as it directly enables tuning of their mechanical and optical properties. Decades of research have shown that polymer structure and processing conditions play key roles in influencing their crystallization morphology and kinetics.^3,5^ For example, industrial processes commonly employ fast cooling rates combined with extensional or shear flows to modulate the crystalline morphology of polyolefins, enabling precise tuning of their resulting macroscopic properties and product performance.^5–9^ Therefore, understanding the non-isothermal crystallization behavior of POs is essential for informing rational process design and tailoring materials to address application-specific needs.

While plastics are essential to modern life, their improper disposal presents significant environmental and health risks. In recent decades, considerable efforts have focused on addressing the sustainability challenges of plastics, including the design of next-generation materials with end-of-life considerations and improved strategies for managing existing plastic waste. Notably, the emergence of dynamic polymer networks offers a promising materials solution enabled by reversible crosslinking chemistries.^10–15^ Specifically, the term covalent adaptable network (CAN) further describes these materials, which can be divided into the categories of “dissociative” and “associative” networks based on the bond exchange mechanism. When CANs are reprocessable and undergo associative bond exchange while retaining their crosslinking density, they can be specifically classified as vitrimers.

PO-derived vitrimers are an emerging research area where a variety of chemistries have been demonstrated, including transesterification,^16–18^ disulfide exchange,^19–26^ boronic ester transesterification/dioxaborolane metathesis,^27–31^ and many others.^32–37^ These materials offer the potential to enhance functionalities and extend the material lifetime while maintaining comparable mechanical performance. Notably, most of the literature focuses on the development of vitrimer chemistry and explores how dynamic crosslinks, catalysts, and reprocessing conditions affect the macroscopic properties of these materials, including their thermal, mechanical, and flow behaviors. Additionally, there have been significant efforts to understand the bond exchange and network rearrangement behaviors in the context of thermal transitions (e.g., topological freezing temperature),^22,32,34,38^ energy dissipation/stress relaxation^39–44^ and creep resistance,^21,25,29,45–47^ allowing material design strategies toward more robust networks. In particular, the impact of catalyst identity on dynamic network relaxation behaviors is a complicated topic and an ongoing research area. Notably, works from Torkelson et al.^20,25,26,48^ found that in PO-derived CANs, the relaxation behaviors of the materials were largely determined by α-relaxation of polymer chains, rather than dynamic bond exchange, potentially attributed to the low crosslink densities found in a typical PO-derived system. For semicrystalline vitrimers, these systems are complicated due to the potentially competing effects and interplay of crystallization and crosslinking. For example, studies have shown that the degree of crystallinity, along with crystal spherulite and lamellae size, is directly related to the thermal and mechanical properties of the material, but crosslinking can limit polymer chain mobility, causing a reduction in the overall degree of crystallinity. Moreover, Ricarte et al.^30^ observed macro- and microphase separations in PE-derived vitrimers due to incompatibilities between the PE backbone and dioxaborolane maleimide crosslinkers. While many studies examine how network formation influences the material properties of PO-derived vitrimers via elucidating structure–property relationships, there remains a limited understanding of how dynamic crosslinking and catalyst effects impact their crystallization behaviors. In particular, the roles of both the cross-linked and non-crosslinked regions in governing overall crystallization behavior and resulting crystal morphology are underexplored, yet this knowledge is critical for the continued advancement and design of PO-derived vitrimers.

This study utilizes diglycidyl ether bispheol A (DGEBA) as a crosslinker and linear low-density PE grafted with maleic anhydride (PE_MA_) to produce model semicrystalline PO-derived vitrimers. The focus is on understanding how dynamic cross-links and catalysts influence vitrimer crystallization. In addition to analyzing the full vitrimeric material, both the insoluble gel fraction and the soluble phase fraction were examined to understand the impacts caused by having a mixed component system. Specifically, this work uses nonisothermal conditions to study crystallization mechanisms and growth kinetics with the Jeziorny-modified Avrami and Kissinger models. It was found that both crosslink formation and the catalyst affect the crystallization behaviors. While network formation can reduce crystal growth rates, the presence of a catalyst also influences crystallization activation energies, depending on whether the specific cooling rate ranges are nucleation or growth-dominated. This study provides important insights for the development of sustainable polymeric materials associated with semicrystalline POs derived dynamic networks.

## Experimental Section

### Materials

Linear low-density polyethylene (LLDPE) was obtained from ASTM as part of their proficiency testing program. TYMAX maleated LLDPE, grade GT4300 (PE_MA_), has a maleic anhydride (MA) content of approximately 1.5 wt % (i.e., 0.43% of monomers contain MA), as confirmed by titration in a previous study, and was obtained from Westlake Chemical. Bisphenol A diglycidyl ether (DGEBA), zinc stearate (ZnSt), and 1,5,7-triazabicyclo[4.4.0]­dec-5-ene (TBD) were purchased from Sigma-Aldrich. Xylenes (ACS Reagent) and methanol (HPLC grade) were obtained from Fisher Chemical. All chemicals were used as received.

### Sample Preparation

PE_MA_-DGEBA_Zn_, PE_MA_-DGEBA_Zn_-insol, PE_MA_-DGEBA_TBD_, and PE_MA_-DGEBA_TBD_-insol vitrimer samples were compounded using an Xplore MC5 microcompounder with a screw speed of 20 rpm at 200 °C (for samples with ZnSt) and 170 °C (for samples with TBD) with a recirculation time of 10 min. The recirculation time was determined using the force output, which is related to the material’s viscosity (Figure S1). For PE_MA_-DGEBA_Zn_, formulations consisted of PE_MA_ (2.5 g) and DGEBA (65 mg, 1:1 ratio of epoxy: MA), and ZnSt (25 mg or 1 wt % of PE_MA_), and all components were introduced to the compounder at the same time. To make PE_MA_-DGEBA_Zn_-insol, the soluble portion of PE_MA_-DGEBA_Zn_ was first extracted by submerging the sample in hot xylenes at 120 °C for ∼24 h. Subsequently, the xylenes were removed by a pipet while still hot, and the sample was vacuum-dried at 60 °C for ∼48 h to yield PE_MA_-DGEBA-insol, as the catalyst, ZnSt, could also be removed during the extraction. The soluble portion, PE_MA_-DGEBA-sol, was collected by precipitating the hot xylenes into MeOH and gravity filtering, followed by vacuum drying. To add the catalyst back into the system and form PE_MA_-DGEBA_Zn_-insol, PE_MA_-DGEBA-insol (1.25 g) and ZnSt (12.5 mg or 1 wt % of PE_MA_-DGEBA-insol) were compounded with both components introduced to the compounder at the same time. For PE_MA_-DGEBA_TBD_, formulations contained PE_MA_ (1.5 g) and DGEBA (39 mg, 1:1 ratio of epoxy: MA), and TBD (15 mg or 1 wt % of PE_MA_), and all components were introduced to the compounder at the same time. To compound PE_MA_-DGEBA_TBD_-insol, the soluble fraction was removed in the same way described above, and batches were done with 2 g of PE_MA_-DGEBA-insol and TBD (∼20 mg or 1 wt % of PE_MA_-DGEBA-insol).

In addition to the vitrimer samples, blends of PE_MA_ and catalysts were made compounding with a screw speed of 100 rpm and temperatures of 200 °C (ZnSt) and 170 °C (TBD) with recirculation times of 5 min. Batches consisted of PE_MA_ (3.0 g) and either ZnSt or TBD (∼30 mg or 1 wt %) with both components added at the same time.

Samples for DMA measurements were hot pressed by using a Carver 4386 press. Samples were pressed at 170 °C for 15 min, where the first 10 min consisted of heating with no pressure, 3 min under 3 t of pressure, and 2 min under 6 t using polytetrafluoroethylene (PTFE) release films on both sides of the mold (2 cm × 0.5 cm × 0.05 cm). The samples were cooled on an aluminum bench with a steel heat sink, compressing the mold for ∼15 min before the cooled bars were removed from the mold.

### Characterization

Fourier transform infrared (FTIR) spectroscopy was run on a PerkinElmer Frontier spectrometer with an attenuated total reflectance (ATR) sampling accessory. An average of 32 scans was taken for each sample with a resolution of 4 cm^–1^ over a range of 4000–600 cm^–1^. All samples were normalized using the characteristic peak at 2916 cm^–1^ corresponding to the asymmetric CH stretching. Dynamic mechanical analysis (DMA) was performed using a TA Instruments Discovery DMA 850. Data acquisition and analysis were performed using TRIOS software by TA Instruments; all experiments were done using a film tension clamp. The oscillation temperature ramp experiments were done with a strain of 0.05%, a frequency of 1.0 Hz, a preload force of 0.01 N, and a temperature ramp rate of 3 °C/min from 25 to 200 °C. Oscillation strain sweep experiments were performed at 70 and 140 °C with a soak time of 5 min, frequency of 1.0 Hz, a preload force of 0.01 N, and over strains ranging from 0.01 to 5%. Tests were stopped early if the moduli started to deviate from the linear viscoelastic region (LVR). Stress relaxation experiments were performed at 70 and 140 °C with a soak time of 5 min, a preload force of 0.01 N, and a strain of 0.2%. This strain was chosen because it is within the LVR for all samples (Figure S2), and the tests were set up to run for 600 min but were stopped when 90% of the initial stress had relaxed. The relaxation times (τ) reported are the time for each sample to relax to 1/e (∼37%) of the initial stress.

Differential scanning calorimetry (DSC) was run on all samples using a TA Instruments Discovery DSC 250. A sample size of ∼4 to 8 mg was used for all samples. LLDPE and PE_MA_ were run using pellets, vitrimer materials were run with extrudate from the microcompounder, and PE_MA_-DGEBA-sol was run with the powder collected from the extraction. Data acquisition and analysis were performed using TRIOS software by TA Instruments. For non-isothermal crystallization kinetics, the samples were first heated to 200 °C at 10 °C/min and held isothermally for 5 min to erase the thermal history. Then the sample was cooled to 25 °C at cooling rates (Φ) of 2, 5, 10, 20, and 40 °C/min, held at 200 °C for 5 min between each cooling step. Additional cycles with cooling rates of 30 and 50 °C/min were performed for PE_MA_-DGEBA_TBD_.

The degree of crystallinity (χ_c_) was determined using the second heating step, following the 2 °C/min cool, for all samples using the following equation:

$$\chi_{c}=\left(\frac{\Delta H_{f}}{\Delta H_{f}^{o}}\right)\times 100\%$$ 1

where Δ*H*_f_ is the enthalpy of fusion and Δ*H*_f_^o^ is the standard enthalpy of fusion for PE and is equal to 293 J g^–1^.

Crystal lamellar thickness (*L*) and lamellar thickness distributions were also determined using the sample’s second heating step. The Gibbs–Thomson eq (eq 2) was used to calculate *L* and eq 3 describes the thickness distributions.

$$T_{m}=T_{m}^{o}\left(1-\frac{2\sigma_{e}}{\Delta H_{f,c}^{o}L}\right)\%$$ 2

$$\frac{1}{M}\frac{dM}{dL}=\frac{dE}{dT}\frac{(T_{m}^{o}-T_{m}{)}^{2}\rho_{c}}{2\sigma_{c}T_{m}}\%$$ 3

where *T*_m_ is the peak melting temperature (in K) for a crystalline lamellae thickness, *L*, *T*_m_^o^ is the equilibrium melting temperature of PE (415 K), σ_e_ is the surface energy of the basal surface of a lamellar crystal (6.09 × 10^–6^ J cm^–2^), and Δ*H*_f,c_^o^ is the enthalpy of fusion for the crystalline phase. *M* is the mass of the crystalline phase in the sample, d*E*/d*T* is the amount of energy required to melt the d*M* portion of the crystalline phase, and ρ_c_ is the density of the crystal phase (∼1 g cm^–3^).

Crystallization kinetics were analyzed using the Avrami model, which can be extended to non-isothermal crystallization using the Avrami equation shown below:

$$X\left(t\right)=1-\exp\left(-Kt^{n}\right)$$ 4

or

log[−ln(1−X(t))]=logK+nlogt 5

where *n* is the Avrami exponent and is related to the mode of nucleation and crystal growth geometry, and *K* is the Avrami constant and describes the overall rate of crystallization. *X*(*t*) is the instantaneous relative crystallinity at time *t* and can be calculated by using eq 6.

$$X(t)=\frac{Q_{t}}{Q_{\infty }}=\frac{\int_{0}^{t}\left(\frac{dh}{dt}\right)dt}{\int_{0}^{\infty }\left(\frac{dH}{dt}\right)dt}$$ 6

Here, *Q*_*t*_ is the instantaneous heat flow, *Q*_*∞*_ is the total heat flow throughout the crystallization process, and d*H*/d*t* is the instantaneous enthalpy change rate. For non-isothermal conditions, the crystallization time (*t*) can be calculated using the crystallization temperature (*T*) using the following equation:

$$t=\frac{T_{\mathrm{onset}}-T}{\phi }$$ 7

where *T*_onset_ is the onset temperature of the crystallization process and ϕ is the cooling rate. The crystallization half-times (*t*_1/2_), determined from the *X*(*t*) plots in Figure S3 can be found in Table S1. Due to the crystallization kinetic measurements being non-isothermal, it has been established that *K* should be corrected to obtain the Jeziorny-modified Avrami constant (*K*'**) at a normalized cooling rate and can be determined using eq 8.^49–51^

$$\log K^{\prime }=\frac{\log K}{\phi }$$ 8

The crystallization activation energy was determined using the Kissinger equation below:^52^

$$\ln\left(\frac{\phi }{T_{p}^{2}}\right)=C-\frac{\Delta E}{RT_{p}}$$ 9

where *C* is a constant, *R* is the gas constant (8.314 J/mol), *T*_p_ is the peak crystallization temperature in K, and Δ*E* is the activation energy.

## Results and Discussion

Figure 1a shows the chemical structures of the PE_MA_, which was crosslinked with DGEBA through a ring-opening reaction between the maleic anhydride (MA) and epoxide rings in the presence of zinc stearate (ZnSt) as a catalyst to form vitrimer samples (PE_MA_-DGEBA_Zn_). The presence of ZnSt allows for the formation of ester crosslinks to be reversible and dynamic at elevated temperatures, as illustrated in Figure 1b. More specifically, the zinc salt can coordinate with carbonyl oxygens to increase the electrophilicity, making the ester groups more susceptible to nucleophilic attack from any free alcohols in the system, and this transesterification reaction enables the covalent bonds to become reversible at elevated temperatures. For this work, PE_MA_-DGEBA_Zn_ was prepared by using reactive extrusion with an epoxy: MA ratio of 1:1 with a catalyst loading of 1 wt % relative to the mass of PE_MA_. Commodity LLDPE without MA was included in this study, where PE_MA_ and the LLDPE were nearly identical in all analyses described below, and the data can be found in the Supporting Information.

**Figure 1:**
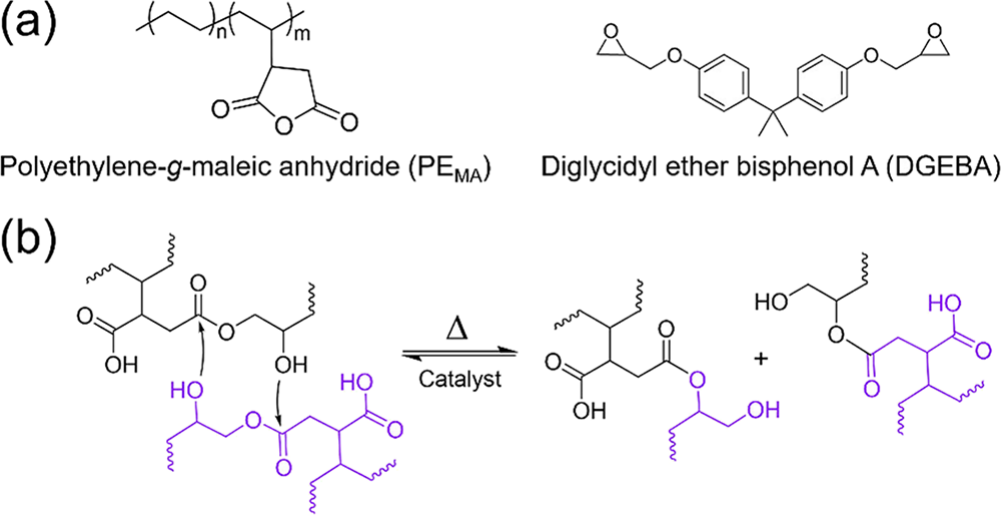
(a) Chemical structures of PE_MA_ and the crosslinker, DGEBA, used to make the dynamic networks and illustration of the dynamic chemistry. (b) Vitrimer transesterification reaction catalyzed through Lewis acid activation.

Fourier transform infrared (FTIR) spectroscopy was used to monitor the progress of the reaction (Figures 2a and S4). Characteristic bands for PE can be observed in both the neat PE_MA_ and vitrimer sample at 2916 and 2850 cm^–1^ from asymmetric and symmetric C–H stretching, respectively. Additionally, the band at 1473 cm^–1^ is due to CH_2_ scissoring motions, and the band at 719 cm^–1^ is associated with CH_2_ rocking. To gain more insight into the formation of ester bonds, a zoomed-in plot with the wavenumber region corresponding to carbon–oxygen bonds (∼1450 to 1850 cm^–1^) can be found in Figure 2b. The band at 1790 cm^–1^ corresponds to C=O stretching from the ring-closed MA (grafted on the PE_MA_). The band at 1712 cm^1^ is from C=O stretching of carboxylic acids, resulting from the ring-opened form of MA. The reduction in intensity of both of these bands (1790 and 1712 cm^–1^) along with the emergence of the band at 1740 cm^–1^ corresponding to C=O stretching of the formed ester in the PE_MA_-DGEBA_Zn_ spectrum, indicates the formation of crosslinking. Furthermore, the presence of DGEBA in the vitrimer sample can be validated by the band at 1510 cm^–1^ from the aromatic C–C stretching.

**Figure 2:**
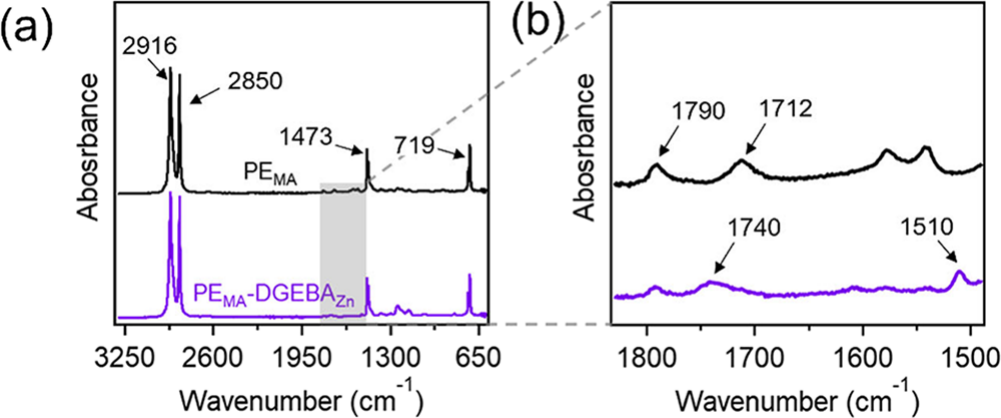
(a) FTIR spectra (normalized to the C–H band at 2916 cm^–1^) of PE_MA_ and PE_MA_-DGEBA_Zn_ and (b) close-up of the FTIR spectra in the range highlighting the esterification reaction.

Differential scanning calorimetry (DSC) was performed to understand the impact of dynamic crosslinking on the crystallization and melting behaviors of these samples. Figure 3a shows the melting events for both PE_MA_ and PE_MA_-DGEBA_Zn_. The melting endotherm of neat PE_MA_ has a single peak at 122 °C and a degree of crystallinity (χ_c_) of 41.3%, indicating that there is a single crystallite size population. However, the endotherm for PE_MA_-DGEBA_Zn_ is bimodal with peaks at 111 and 122 °C and exhibits a χ_c_ of 32.2%, suggesting two mixed components within the sample. Informed by our previous study, these can be allocated to the crosslinked and un-crosslinked phases within the vitrimer sample.^53^ The higher melting temperature (*T*_m_) of the vitrimer is very similar to the *T*_m_ of neat PE_MA_, indicating that this crystallite population is most likely from the un-crosslinked phase. The decrease in the overall χ_c_ of the vitrimer can be attributed to the crosslinking hindering the growth of crystallites, as well as a slight decrease in the amount of PE in the system due to the presence of the DGEBA-based cross-linker.

**Figure 3:**
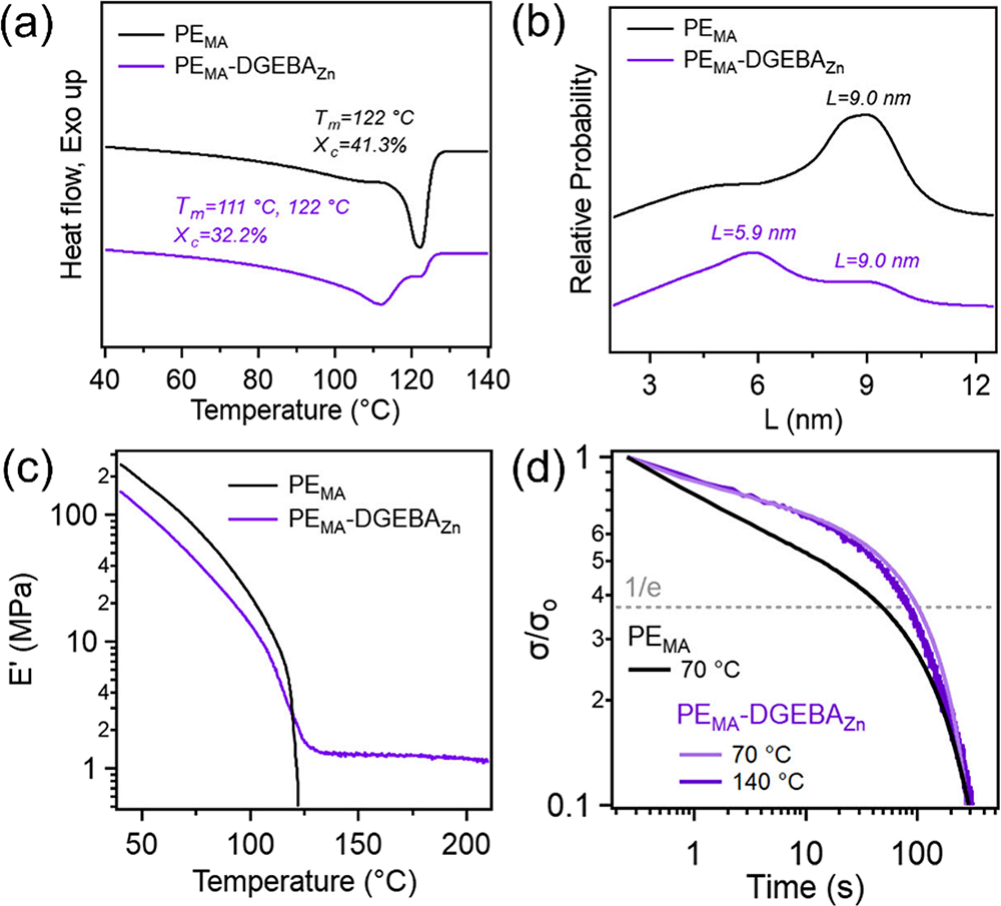
(a) DSC thermograms highlighting *T*_m_ and χ_c_, (b) relative probabilities of the lamellar thickness for PE_MA_ and PE_MA_-DGEBA_Zn_, (c) storage modulus–temperature plot, and (d) stress relaxation at 70 and 140 °C of PE_MA_ and PE_MA_-DGEBA_Zn_.

Additionally, the crystallite lamellar thickness (*L*) and its distribution can be directly calculated using the DSC heat thermograms and eqs 2 and 3. The lamellar thickness distributions for bulk neat PE_MA_ and PE_MA_-DGEBA_Zn_ can be found in Figure 3b. It can be seen that PE_MA_ has a single crystallite population with lamellar thicknesses ranging from ∼7 to 10 nm with a peak at 9.0 nm. However, once crosslinked to form the vitrimer, two distinct *L* size populations emerge with peaks at 5.9 and 9.0 nm. The population with an *L* of 9.0 nm is attributed to the un-crosslinked portion of the vitrimer sample (consistent with the PE_MA_ sample) and to the decreased *L* of the crosslinked portion. This reduction in thickness can be associated with network formation where the crosslinks can hinder crystal formation, and this has been previously reported with other PE-derived vitrimer systems.^19,22,53^

To further demonstrate crosslink formation, dynamic mechanical analysis (DMA) was run on the samples, monitoring the storage modulus (*E'*) at elevated temperatures over a range of 40–225 °C (Figures 3c and S5). The initial decrease in *E*'** for the vitrimer sample (110.4 MPa at 50 °C) compared to neat PE_MA_ (185.4 MPa at 50 °C) is attributed to the reduced χ_c_ below *T*_m_, which is consistent with the DSC results.^54–56^ The melting behaviors of the samples can be observed by the characteristic drop of *E*'** at elevated temperatures, where the onset for the modulus drop is at 116 °C for PE_MA_ and 106 °C for PE_MA_-DGEBA_Zn_. These temperatures align with the *T*_m, onset_ from the DSC thermograms, which are 114 and 96 °C for neat PE_MA_ and the vitrimer sample, respectively. The end-set temperature for the *E*'** drop and melting for PE_MA_-DGEBA_Zn_ is 126 °C (125 °C from DSC data). However, the end-set *T*_m_ for PE_MA_ cannot be determined with DMA, due to the material flowing above 120 °C. PE_MA_-DGEBA_Zn_ has a rubbery plateau above *T*_m_ with an *E'* of 1.3 MPa, indicative of network formation.

Additional DMA experiments were run on the samples to understand how cross-linking impacted the stress relaxation behaviors at 70 and 140 °C with a strain of 0.2% in tension (within linear viscoelastic region for all samples, Figure S6). These temperatures were chosen to have one temperature with intact crystallites, 70 °C, as this is ∼25 to 50 °C below *T*_m, onset_ (from DSC) for both samples, and a temperature where the crystals are melted, 140 °C (as seen in both DSC and DMA). In Figures 3d and S6, it can be observed that PE_MA_ relaxes significantly faster than the vitrimer sample at 70 °C. Sample relaxation time (τ) can be determined by monitoring the time it takes to relax to 1/e (∼37%) of the initial stress and was found to be 52.3 s for PE_MA_ at 70 °C and 101 and 83.3 s for PE_MA_-DGEBA_Zn_ at 70 and 140 °C, respectively. The increase in τ for PE_MA_-DGEBA_Zn_ for both temperatures was anticipated as network formation is known to restrict molecular motion, causing energy to dissipate more gradually. These results (Table S2) further confirm the network formation within our PE_MA_-DGEBA_Zn_, and the introduction of crosslinking has a clear impact on the PE dynamics.

To understand the crystallization mechanisms and kinetics, nonisothermal conditions were used because they more closely resemble industrial processing conditions, which are crucial to the final material properties and product performance.^57^ The nonisothermal crystallization thermograms of PE_MA_ and PE_MA_-DGEBA_Zn_ are shown in Figure 4a,b, respectively, with cooling rates (Φ) of 2, 5, 10, 20, and 40 °C/min. Here, we see that as the cooling rate increases, both the onset (*T*_onset_) and peak (*T*_p_) crystallization temperatures decrease (summarized in Table S1). Additionally, the crystallization peaks become broader with increasing cooling rates due to imperfect polymer crystal structures being formed under the more rapid cooling.^58^ Similar to the endotherms shown previously, PE_MA_-DGEBA_Zn_ exotherms are bimodal, where the higher *T*_p_, *T*_p,1_ can be attributed to the un-crosslinked portion and the lower, *T*_p,2_, to the crosslinked portion of the vitrimer. At the same cooling rates, both *T*_p,1_ and *T*_p,2_ show lower *T*_p_ values than those of neat PE_MA_, suggesting that a greater degree of supercooling would be required for crystallization upon crosslinking, due to the hindrance of chain mobility and packing.

**Figure 4:**
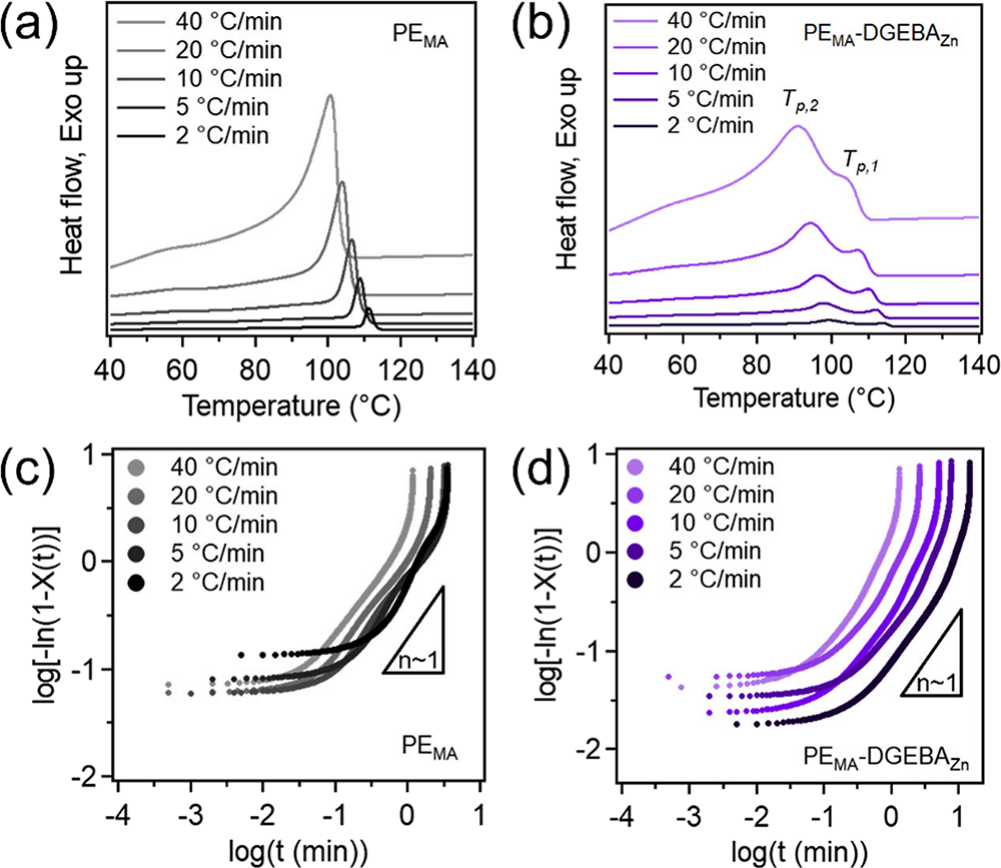
DSC thermograms with various cooling rates and Avrami plots of (a, c) PE_MA_ and (b, d) PE_MA_-DGEBA_Zn_.

To further analyze the nonisothermal crystallization, the Avrami model was applied for both PE_MA_ and PE_MA_-DGEBA_Zn_. The Avrami model, which describes material transformation from one phase to another with a constant temperature, has been widely utilized to study isothermal crystallization of semicrystalline polymers.^59,60^ This method can be extended to nonisothermal crystallization conditions with the assumption that the system has a constant crystallization temperature.^7,50,57,58,61–63^ The Avrami equation and a rearranged form used to create the Avrami plots can be found in the Experimental Section (eqs 4 and 5). Additionally, eq 6 was utilized to determine the instantaneous relative crystallinity (*X*(*t*)) and eq 7 to convert the temperatures to the crystallization time (*t*). The Avrami plots for PE_MA_ and PE_MA_-DGEBA_Zn_ can be found in Figure 4c, d, respectively. In these plots, it can be observed that at early times, the crystallization behavior of these samples was dominated by nucleation without significant crystal growth with slopes ≪ 1. In the second region, the main crystallization phase, there is both nucleation and crystal growth, and this is the region used for further analysis described below. The third region, slope ≫ 1, corresponds to late stages of crystallization, which is not well-described by the Avrami equation.

From the Avrami plots, both the Avrami exponent (*n*) and the Avrami constant (*K*) can be extracted using the linear region that the Avrami equation adequately describes using the slope and *y*-intercept (eq 5), respectively. The Avrami exponent is related to the mode of nucleation, type of growth process, and crystal growth geometry, while the Avrami constant provides the overall rate of crystallization. However, due to the crystallization being done with nonisothermal conditions, it has been established that *K* should be corrected to the Jezirony-modified Avrami constant (*K*'**) using eq 8.^49,51,58^ Literature reports a broad range of Avrami parameters for polymers, and this can be due to several factors, including the factor that the Avrami linear domain is not clearly defined. Additionally, polymer molecular weight, sample mass, and the presence of additives can all affect the crystallization process. Previous studies have reported Avrami exponents for LLDPE in the range from 2 to 4.^57,64–66^ The Avrami parameters for the second (main) crystallization phase (*n*_2_ and *K*'**_2_) for PE_MA_ and the vitrimer sample can be found in Figure 5. In Figure 5a, for both the neat PE_MA_ and the vitrimer, *n*_2_ ranges around 1 for all cooling rates, implying 1D growth with heterogeneous nucleation behaviors on surfaces or impurities. However, since nonisothermal conditions were used, it is understood that these *n* and *K* values may be different from those derived from isothermal processes.^57,67,68^ It is observed that PE_MA_ has a slight dependence on the cooling rate, particularly at the slower rates (2 and 5 °C/min), whereas the PE_MA_-DGEBA_Zn_ stays consistent across all cooling rates at a slightly decreased value. The *K*'**_2_ values for both samples can be found in Figure 5b. Both samples show an increase in the rate of crystallization with an increase in the cooling rate, with the trend being more pronounced in the vitrimer sample. The vitrimer samples exhibit reduced *K*'**_2_ values, particularly at slower cooling rates (2–10 °C/min), which is attributed to crosslinking restricting chain mobility and thereby slowing crystallite growth compared to neat PE_MA_. As the cooling rate increases to approximately 20–40 °C/min, the *K*'**_2_ values begin to converge. This convergence is likely due to enhanced supercooling at higher cooling rates, which promotes crystallization at lower temperatures. Under these conditions, chain mobility is further limited, favoring nucleation-driven crystal growth and resulting in a plateau in *K*'**_2_. Given the relatively low MA content in the PE samples, the effects of fast cooling-induced mobility restriction at higher cooling rates can surpass those of cross-linking, leading to the observed convergence in crystallization behavior.

**Figure 5:**
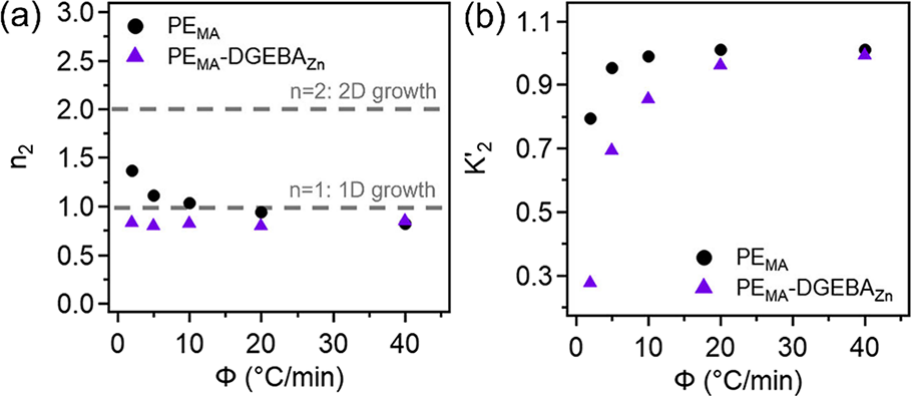
(a) Avrami exponents and (b) Jeziorny-modified Avrami constants for PE_MA_ and PE_MA_-DGEBA_Zn_ from the second region of their corresponding Avrami plots.

While understanding the crystallization behaviors of the entire vitirmeric material is important, it is also necessary to study the individual components (soluble/non-crosslinked and insoluble/crosslinked) of the material to get a more complete picture. Particularly, previous studies observed macro- and microphase separation in their PO-derived vitrimers,^30^ and another study found their overall PE vitrimer mechanical properties were intermediate to the individual soluble and insoluble portions of the material.^53^ Moreover, to separate the material components, the soluble, non-crosslinked fraction of the vitrimer material was removed through solvent extraction in 120 °C xylenes. The soluble fraction (PE_MA_-DGEBA-sol) could be recovered by crashing it out into methanol, while the insoluble gel fraction (PE_MA_-DGEBA_Zn_-insol) remained once the xylenes were removed.

Once the soluble and insoluble portions were obtained, the same characterizations as outlined for PE_MA_ and PE_MA_-DGEBA_Zn_ were performed to identify the presence of crosslinks (FTIR and DMA), and can be found in Figures S4 and S5. The results showed that the soluble fraction contained only a limited amount of maleic anhydride (MA) and no detectable DGEBA, while the insoluble fraction remained a network with DGEBA present. These results confirm that our method can selectively extract distinct components from the vitrimer system. The impact of cross-linking on the stress relaxation behaviors can be observed for PE_MA_-DGEBA_Zn_-insol, where at 70 °C, τ is 121 s and at 140 °C, τ is 72.0 s (Figure S6). The vitrimer components then went through the same nonisothermal crystallization conditions as previously described, and their crystallinity development and Avrami plots can be found in Figures S7 and S8, respectively; the value of Avrami exponents and constants are shown in Figure S9. From these data, it was observed that both the soluble and insoluble portions had *n*_2_ values remaining around 1, indicating that the crystal growth mechanism/geometry was not majorly impacted. In addition, the soluble phase had *K*'**_2_ values more similar to PE_MA_, while the insoluble portion remained slightly more suppressed at 2 °C/min, with both portions showing limited dependence on cooling rates of ≥10 °C/min (∼40 °C/min for the full vitrimer material).

The crystallization half-time (*t*_1/2_) values, determined using the *X*(*t*) plots, can be found in Figure 6a. A trend similar to that seen in the *K*'** values is observed, where crystal growth rates are increasingly suppressed with decreasing cooling rates for the vitrimer. Here, this is confirmed by PE_MA_-DGEBA_Zn_ having an increased *t*_1/2_ when compared to those of neat PE_MA_ and PE-DGEBA-sol. Once separated, PE_MA_-DGEBA-sol has nearly identical *t*_1/2_s to neat PE_MA_, highlighting the impact that the cross-links have on crystallite growth rates.

**Figure 6:**
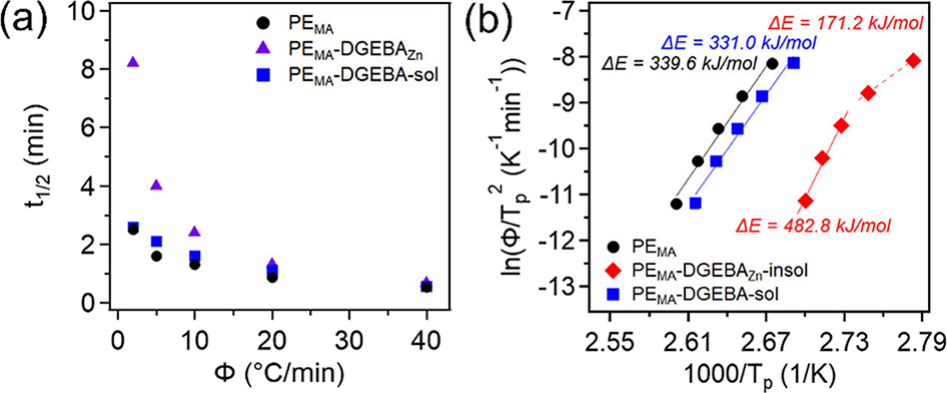
(a) *t*_1/2_ for PE_MA_, PE_MA_-DGEBA_Zn_, and PE_MA_-DGEBA-sol with various cooling rates. (c) ln­(Φ/*T*_p_^2^) vs 1000/*T*_p_ for the different components of PE_MA_-DGEBA_Zn_.

The Kissinger method was used to determine the crystallization activation energies (Δ*E*) of neat PE_MA_ as well as the soluble and insoluble components of the vitrimer. This was done using eq 9 which relies on the specific cooling rates and crystallization peak temperatures (*T*_p_). In general, Δ*E* is made of the nucleation activation energy (free energy of formation of crystal nuclei with a critical size) and transport activation energy (transport polymer segments across the phase boundary to the crystal growth surface).^69^ The resulting plot is found in Figure 6b, where Δ*E* can be determined using the slope. From this plot, it can be seen that the activation energies from PE_MA_ and PE_MA_-DGEBA-sol are very similar to values of 339.6 and 331.0 kJ/mol, respectively. Interestingly, the insoluble fraction shows two distinct regions with different activation energies. The region consisting of cooling rates from 2 to 10 °C/min has an Δ*E* of 482.8 kJ/mol, while the faster cooling rates, 20–40 °C/min, have a value of 171.2 kJ/mol. The behavior of the insoluble fraction indicates that the ZnSt is likely acting as a nucleating agent. At the faster cooling rates, crystallization appears to be dominated by nucleation, where the presence of ZnSt could lower the overall Δ*E* by decreasing the nucleation activation energy. However, at the slower cooling rates, crystallization is dominated by growth, and the crosslinks would start to have a higher impact on Δ*E* by hindering chain mobility and thus increasing the transport activation energy.

To confirm the impacts of the catalyst, a blend of PE_MA_ with 1 wt % ZnSt was formulated. The DSC thermograms, development of crystallinity, and Avrami plot for the ZnSt blend can be found in Figures S7and S8. In Figure 7a, it can be seen that while slightly decreased from neat PE_MA_, the *n*_2_ values remain ∼1, meaning the crystallization mechanism and geometry are not impacted by the presence of the ZnSt. The Kissinger method was applied using the *T*_p_s and cooling rates, and the resulting plot is shown in Figure 7b. Here, we observe the emergence of two distinct regions, where at the faster cooling rates, the Δ*E* is 173.8 kJ/mol, very similar to that of PE_MA_-DGEBA_Zn_-insol. We note that there is still an increase in Δ*E* at the slower cooling rates, Δ*E* = 380.0 kJ/mol, over that of neat PE_MA_, indicating there is another mechanism acting to increase the activation energy in this region beyond just the presence of crosslinks in the vitrimer sample. This is attributed to the increase in nucleation density due to the addition of ZnSt, which can act as a nucleating agent, where the increase in nucleation density causes the crystallites to crowd each other, causing the chains to have lowered mobility, thus increasing the transport activation energy.

**Figure 7:**
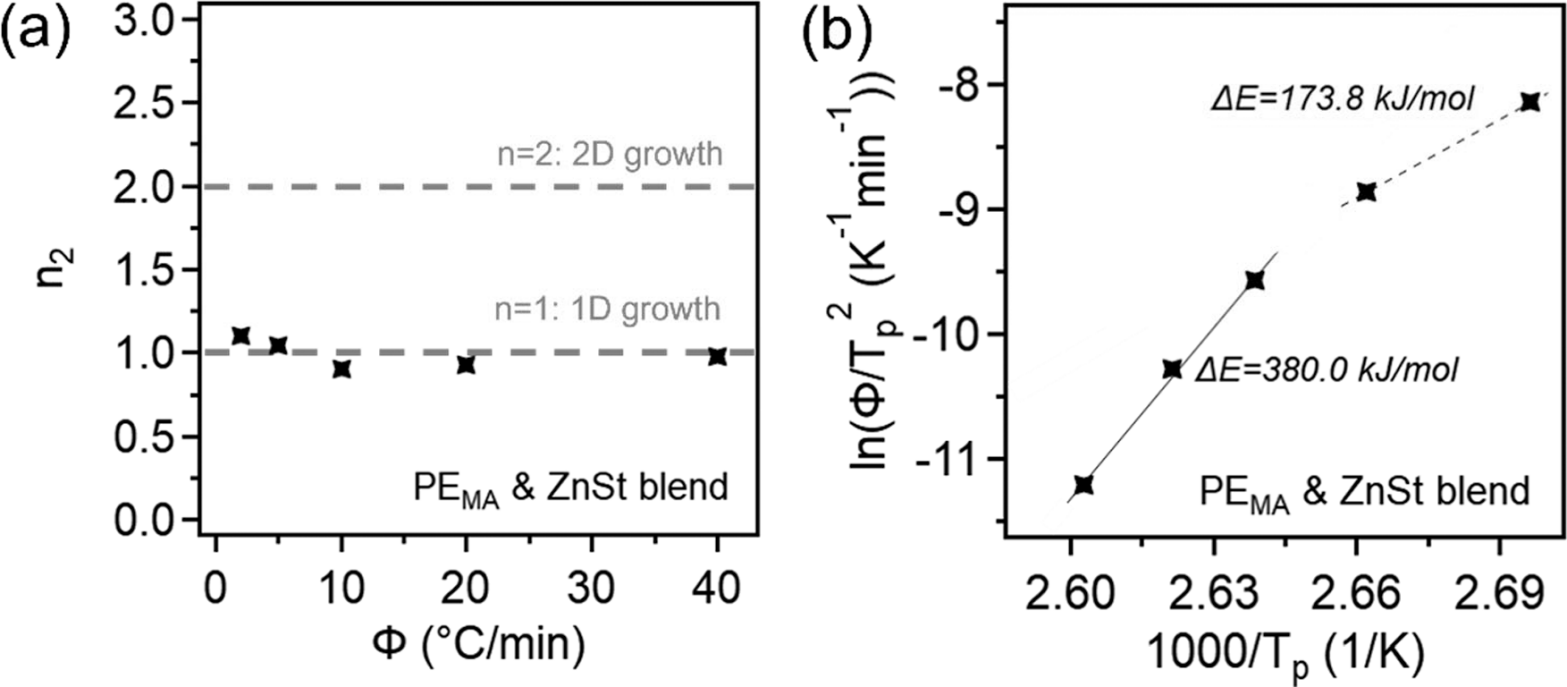
(a) Avrami exponents over various cooling rates and (b) ln­(Φ/*T*_p_^2^) vs 1000/*T*_p_ for PE_MA_ blended with 1 wt % ZnSt.

To further understand the influences of dynamic crosslinking and catalysts on crystallization behaviors in the PO-derived vitrimers, an additional system was prepared using another common catalyst for transesterification, 1,5,7-triazabicyclo[4.4.0]­dec-5-ene (TBD). Similarly, these materials were compounded using PE_MA_ and DGEBA (1:1 MA: epoxy), with the presence of 1 wt % (to PE_MA_) TBD to create PE_MA_-DGEBA_TBD_. A physical blend of PE_MA_ and 1 wt % TBD was also made. FTIR and DMA data for PE_MA_-DGEBA_TBD_ can be found in Figures S4 and S5. From this data, we can confirm the formation of crosslinks and presence of DGEBA as well as network formation with a rubbery plateau of ∼1.2 MPa, suggesting a similar cross-link density to its ZnSt counterpart. The DSC thermograms, development of crystallinity, and Avrami plot for the TBD vitrimer and blend are shown in Figures S7and S8. Figure 8a shows that *n*_2_ remains ∼1 for all cooling rates, indicating that TBD does not significantly alter the crystallite growth mechanism or geometry.

**Figure 8:**
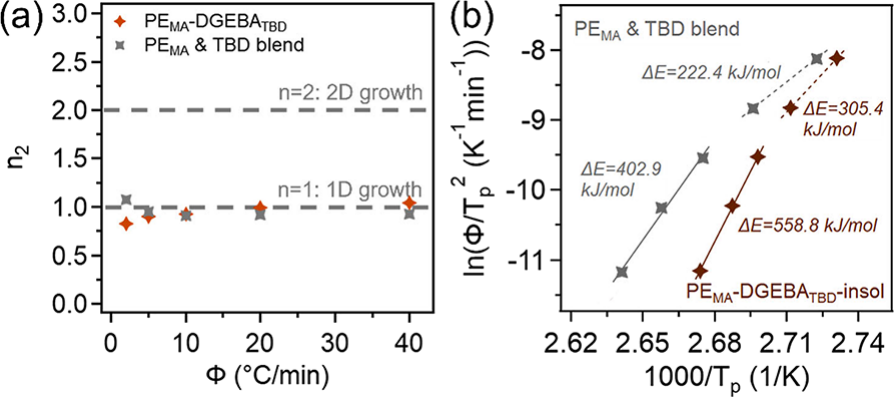
(a) Avrami exponents over various cooling rates and (b) ln­(Φ/*T*_p_^2^) vs 1000/*T*_p_ for PE_MA_-DGEBA_TBD_ and PE_MA_ blended with 1 wt % TBD.

To investigate the impacts of TBD on the crystallization activation energy, the cross-linked portion of the TBD vitrimer was separated using the same hot xylene solvent extraction technique previously described. The FTIR trace for PE_MA_-DGEBA_TBD_-insol is shown in Figure S4 where the material shows the presence of ester crosslinks and DGEBA. When the nonisothermal DSC process performed on the insoluble fraction was analyzed, it was observed that some of the soluble portion (higher *T*_p_) remained. However, it appears that a significant amount of the soluble fraction was removed, indicated by the decrease in the crystallization peak corresponding to the uncross-linked fraction as well as the *T*_p,2_ values shifting toward lower temperatures (less influence from the soluble fraction). The Kissinger method was then applied to PE_MA_-DGEBA_TBD_-insol and the PE_MA_ and TBD blend, and the plot is shown in Figure 8b. We observe similar results where TBD lowers Δ*E* (compared to PE_MA_) in the faster cooling rate regions, and the Δ*E*s are 305.4 and 222.4 kJ/mol for the vitrimer and blend, respectively. When examining the slower cooling rate region, we see that the TBD catalyst results in an increased Δ*E* (compared to PE_MA_), where here the Δ*E*s are 558.8 kJ/mol for the vitrimer and 402.9 kJ/mol for the blend. It is found that TBD can also nucleate crystallization, lowering the activation energy in the cooling rate regions dominated by nucleation while increasing the activation energy in cooling rate regions dominated by crystal growth due to increased nucleation density, which can be further increased by cross-linking. Collectively, these results demonstrate that the identity of the catalyst can also significantly influence the activation energy for both nucleation- and growth-dominated crystallization behavior in PE-derived vitrimer systems.

Another interesting observation was made when considering the degree of crystallinity of the materials (listed in Table S1). Considering only the PE portion of the samples, the χ_c, PE_ for PE_MA_-DGEBA_Zn_ across all cooling rates stayed consistent at ∼33% with its TBD counterpart at ∼34%. This decrease in crystallinity from the ∼40% of neat PE_MA_ was originally solely attributed to the crosslinking. However, the blends also have a reduced χ_c, and PE_ the ZnSt blend remains consistent at ∼36% and the TBD blend at ∼34%. Demonstrating that the catalyst being present also reduces the crystallinity in the system, most likely due to the increase in nucleation density that hinders chain mobility observed during the activation energy studies. It is important to note that the graft density of the MA on the PE chain is only 1.5 wt % (corresponding to ∼230 PE repeat units for every MA containing monomer) implying that the crosslink density is likely low, thus the impacts of the catalyst on the resultant degree of crystallinity may not be as apparent in systems with higher grafting densities.

Although industrial cooling rates (which can be up to thousands of °C/min) far exceed those achievable with traditional DSC, the PE_MA_-DGEBA vitrimer results indicate that at faster cooling rates (>20 °C/min), the influence of network formation and catalyst presence on crystallization rates is limited. This is supported by the convergence of *K*'**_2_ and consistent trends in *t*_1/2_ across all samples. However, more pronounced effects from crosslinking likely pertain to crystalline morphology, potentially due to catalyst-induced nucleation under the enhanced cooling at faster cooling rates, which could be explored in future works. These findings suggest that dynamic bonding can be incorporated for reprocessability without significantly compromising the crystallization behavior under practical processing conditions.

## Conclusions

In this work, established chemistries were used to prepare model PO-derived vitrimers using PE grafted with maleic anhydride and subsequently crosslinked with DGEBA using zinc stearate or TBD as a catalyst. We study the impacts of dynamic cross-linking on the nonisothermal crystallization kinetics and mechanisms of semicrystalline vitrimers using the Jezirorny-modified Avrami and Kissinger models. All samples displayed primarily one-dimensional growth with heterogeneous nucleation, with influences from crosslinking or catalyst presence. It was observed that network formation reduced the crystal growth rates by inhibiting chain mobility, as demonstrated by both the Jeziorny-modified Avrami constants and crystallization half-times. It was found that both catalysts, ZnSt and TBD, can act as nucleating agents and lower crystallization activation energies in cooling rate ranges that are dominated by nucleation and increase the activation energy in ranges dominated by crystal growth. This increase in activation energy at a slow cooling rate is attributed to a higher nucleation density, which restricts crystal growth by inducing spatial overcrowding. Additionally, both network formation and catalyst presence reduced the overall degree of crystallinity of the PE vitrimer samples. These findings provide fundamental insight into the impacts dynamic crosslinking and catalysts have on the crystallization behaviors of PE-based vitrimers and the continued advancement of semicrystalline PO-based reprocessable networks.

## Supplementary Material



## Data Availability

The data supporting this study are available upon request from the corresponding author.

## Notes

Force output plot from microcompounder during reactive extrusion, oscillatory strain sweep plots, development of crystallinity plots, FTIR spectra, storage modulus–temperature plot, stress relaxation plot, DSC thermograms with various cooling rates, Avrami plots, Avrami exponents with various cooling rates, Jeziorny-modified Avrami constant with various cooling rates, table listing *T*_onset_, *T*_p_, *t*_1/2_, *n*_2_, *K*'**_2_, and χ_c, PE_ for all the samples, and table listing relaxation times (PDF)

## References

[1] Sauter D. W., Taoufik M., Boisson C. (2017). Polyolefins, a Success Story. Polymers (Basel).

[2] Stürzel M., Mihan S., Mülhaupt R. (2016). From Multisite
Polymerization Catalysis
to Sustainable Materials and All-Polyolefin Composites. Chem. Rev..

[3] Galli P., Vecellio G. (2004). Polyolefins: The Most
Promising Large-Volume Materials
for the 21st Century. J. Polym. Sci., Part A:
Polym. Chem..

[4] Li C. Y. (2020). The Rise
of Semicrystalline Polymers and Why Are They Still Interesting. Polymer.

[5] Mileva, D. ; Tranchida, D. ; Gahleitner, M. Designing Polymer Crystallinity: An Industrial Perspective. In Polymer Crystallization; John Wiley and Sons Inc, August 1, 2018, DOI: 10.1002/pcr2.10009.

[6] Gahleitner M., Mileva D., Androsch R., Gloger D., Tranchida D., Sandholzer M., Doshev P. (1695). Crystallinity-Based Product Design:
Utilizing the Polymorphism of Isotactic PP Homo-and Copolymers. AIP Conf. Proc..

[7] Müller A. J., Arnal M. L., Spinelli A. L., Cañizales E., Puig C. C., Wang H., Han C. C. (2003). Morphology
and Crystallization
Kinetics of Melt Miscible Polyolefin Blends. Macromol. Chem. Phys..

[8] Cherukupalli S. S., Gottlieb S. E., Ogale A. A. (2005). Real-Time
Raman Spectroscopic Measurement
of Crystallization Kinetics and Its Effect on the Morphology and Properties
of Polyolefin Blown Films. J. Appl. Polym. Sci..

[9] Oliveira M.
J., Cramez M. C. (2001). Rotational
Molding of Polyolefins: Processing, Morphology,
and Properties. J. Macromol. Sci. Phys..

[10] Zou W., Dong J., Luo Y., Zhao Q., Xie T. (2017). Dynamic Covalent
Polymer Networks: From Old Chemistry to Modern Day Innovations. Adv. Mater..

[11] Van
Zee N. J., Nicolaÿ R. (2020). Vitrimers: Permanently Crosslinked
Polymers with Dynamic Network Topology. Prog.
Polym. Sci..

[12] Zheng N., Xu Y., Zhao Q., Xie T. (2021). Dynamic Covalent Polymer Networks:
A Molecular Platform for Designing Functions beyond Chemical Recycling
and Self-Healing. Chem. Rev..

[13] Maes S., Badi N., Winne J. M., Du Prez F. E. (2025). Taking Dynamic Covalent
Chemistry out of the Lab and into Reprocessable Industrial Thermosets. Nat. Rev. Chem..

[14] Webber M. J., Tibbitt M. W. (2022). Dynamic and Reconfigurable Materials
from Reversible
Network Interactions. Nat. Rev. Mater..

[15] Ahmadi M., Hanifpour A., Ghiassinejad S., Van Ruymbeke E. (2022). Polyolefins
Vitrimers: Design Principles and Applications. Chem. Mater..

[16] Wang S., Ma S., Qiu J., Tian A., Li Q., Xu X., Wang B., Lu N., Liu Y., Zhu J. (2021). Upcycling
of Post-Consumer Polyolefin Plastics to Covalent Adaptable Networksvia
in Situcontinuous Extrusion Cross-Linking. Green
Chem..

[17] Kar G. P., Saed M. O., Terentjev E. M. (2020). Scalable Upcycling of Thermoplastic
Polyolefins into Vitrimers through Transesterification. J. Mater. Chem. A Mater..

[18] Gao Y., Niu H. (2024). Polypropylene-Based
Transesterification Covalent Adaptable Networks
with Internal Catalysis. Polym. Chem..

[19] Fenimore L.
M., Chen B., Torkelson J. M. (2022). Simple Upcycling of Virgin and Waste
Polyethylene into Covalent Adaptable Networks: Catalyst-Free, Radical-Based
Reactive Processing with Dialkylamino Disulfide Bonds. J. Mater. Chem. A Mater..

[20] Chen B., Debsharma T., Fenimore L. M., Wang T., Chen Y., Purwanto N. S., Torkelson J. M. (2024). Rapidly Self-Healable and Melt-Extrudable
Polyethylene Reprocessable Network Enabled with Dialkylamino Disulfide
Dynamic Chemistry. Macromol. Rapid Commun..

[21] Suazo M. J., Fenimore L. M., Barbon S. M., Brown H., Auyeung E., Cespedes G., Li Pi Shan C., Torkelson J. M. (2024). Extrudable
and Highly Creep-Resistant Covalent Adaptable Networks Made from Polyethylene
and Ethylene/1-Octene Copolymers by Reactive Processing with Aromatic
Disulfide Cross-Links. ACS Appl. Polym. Mater..

[22] Montoya-Ospina M. C., Verhoogt H., Ordner M., Tan X., Osswald T. A. (2022). Effect
of Cross-Linking on the Mechanical Properties, Degree of Crystallinity
and Thermal Stability of Polyethylene Vitrimers. Polym. Eng. Sci..

[23] Montoya-Ospina M. C., Verhoogt H., Osswald T. A. (2022). Processing
and Rheological Behavior
of Cross-Linked Polyethylene Containing Disulfide Bonds. SPE Polymers.

[24] Huang Y. W., Suazo M. J., Barbon S. M., Brown H. A., Auyeung E., Li Pi Shan C., Torkelson J. M. (2025). Polypropylene Covalent Adaptable
Networks with Full Cross-Link Density Recovery after Reprocessing:
Development by Free-Radical Reactive Processing with Resonance-Stabilized,
Aromatic Disulfide Cross-Linkers. ACS Macro
Lett..

[25] Bin
Rusayyis M. A., Fenimore L. M., Purwanto N. S., Torkelson J. M. (2023). Reprocessable,
Creep-Resistant Covalent Adaptable Networks Synthesized Using Conventional
Free-Radical Polymerization Conditions with Piperidine-Based and Non-Piperidine-Based
Dynamic Dialkylamino Disulfide Chemistry. Polym.
Chem..

[26] Fenimore L. M., Suazo M. J., Torkelson J. M. (2024). Covalent Adaptable Networks Made
by Reactive Processing of Highly Entangled Polymer: Synthesis-Structure-Thermomechanical
Property-Reprocessing Relationship in Covalent Adaptable Networks. Macromolecules.

[27] Wang W. Y., Zha X. J., Bao R. Y., Ke K., Liu Z. Y., Yang M. B., Yang W. (2021). Vitrimers of Polyolefin
Elastomer
with Physically Cross-Linked Network. J. Polym.
Res..

[28] Zhao Y., Li J., Ma Y., Wang Y., Jiang C., Yan H., Hao R., Qin J., Shi X., Zhang G. (2023). One-Step Reactive Processing
of Vitrimeric Thermoplastic Polyolefin Elastomer with Greatly Improved
Thermo-Mechanical Property. Polymer.

[29] Wang Z., Gu Y., Ma M., Liu Y., Chen M. (2021). Strengthening Polyethylene
Thermoplastics through a Dynamic Covalent Networking Additive Based
on Alkylboron Chemistry. Macromolecules.

[30] Ricarte R. G., Tournilhac F., Leibler L. (2019). Phase Separation and Self-Assembly
in Vitrimers: Hierarchical Morphology of Molten and Semicrystalline
Polyethylene/Dioxaborolane Maleimide Systems. Macromolecules.

[31] Röttger M., Domenech T., Van Der Weegen R., Breuillac A., Nicolaÿ R., Leibler L. (2017). High-Performance Vitrimers
from Commodity
Thermoplastics through Dioxaborolane Metathesis. Science (1979).

[32] Neidhart E. K., Hua M., Peng Z., Kearney L. T., Bhat V., Vashahi F., Alexanian E. J., Sheiko S. S., Wang C., Helms B. A., Leibfarth F. A. (2023). C-H Functionalization of Polyolefins to Access Reprocessable
Polyolefin Thermosets. J. Am. Chem. Soc..

[33] Vialon T., Sun H., Formon G. J. M., Galanopoulo P., Guibert C., Averseng F., Rager M. N., Percot A., Guillaneuf Y., Van Zee N. J., Nicolaÿ R. (2024). Upcycling Polyolefin Blends into
High-Performance Materials by Exploiting Azidotriazine Chemistry Using
Reactive Extrusion. J. Am. Chem. Soc..

[34] Sun H., Vialon T., Guibert C., Casale S., Nicolaÿ R., Van Zee N. J. (2025). Reactive Processing of Polyethylene with Azidotriazine:
From Modified Thermoplastics to Injection Moldable Covalent Adaptable
Networks. ACS Appl. Polym. Mater..

[35] Lee H., Jang Y., Chang Y. W., Lim C. (2024). Covalent Adaptable
Network of Semicrystalline Polyolefin Blend with Triple-Shape Memory
Effect. Polymers.

[36] Shapiro A. J., Brigandi P. J., Moubarak M., Sengupta S. S., Epps T. H. (2024). Cross-Linked
Polyolefins: Opportunities for Fostering Circularity Throughout the
Materials Lifecycle. ACS Appl. Polym. Mater..

[37] Ash S., Sharma R., Shaker M., Patil S., Cheng S., Rabnawaz M. (2024). One-Pot Synthesis of
Robust Silyl Ether-Based HDPE
Vitrimers with Enhanced Performance and Recyclability. Polymer.

[38] Dey I., Muhammed Ajnas N., Rege S. S., Islam S. S., Misra A., Samanta K., Manna K., Bose S. (2023). Does the Varying
Reactivity
in the Transient Polymer Network through Dynamic Exchange Regulate
the Closed-Loop Circularity in Polyolefin Vitrimers?. ACS Appl. Mater. Interfaces.

[39] Mei B., Lin T. W., Sheridan G. S., Evans C. M., Sing C. E., Schweizer K. S. (2022). Structural Relaxation and Vitrification in Dense Cross-Linked
Polymer Networks: Simulation, Theory, and Experiment. Macromolecules.

[40] Ricarte R. G., Shanbhag S. (2024). A Tutorial Review of
Linear Rheology for Polymer Chemists:
Basics and Best Practices for Covalent Adaptable Networks. Polym. Chem..

[41] Barzycki D. C., Ezzeddine D., Shanbhag S., Ricarte R. G. (2025). Linear Viscoelasticity
of Polystyrene Vitrimers: Segmental Motions and the Slow Arrhenius
Process. Macromolecules.

[42] Ricarte R. G., Shanbhag S. (2021). Unentangled Vitrimer
Melts: Interplay between Chain
Relaxation and Cross-Link Exchange Controls Linear Rheology. Macromolecules.

[43] Yang X., Guo L., Xu X., Shang S., Liu H. (2020). A Fully Bio-Based Epoxy
Vitrimer: Self-Healing, Triple-Shape Memory and Reprocessing Triggered
by Dynamic Covalent Bond Exchange. Mater. Des..

[44] Ge S., Evans C. M. (2025). Influence of Segmental
Dynamics on Bond Exchange in
Imine Vitrimers with Different Polymer Backbones and Cross-Linkers. Macromolecules.

[45] Li L., Chen X., Jin K., Torkelson J. M. (2018). Vitrimers
Designed Both to Strongly Suppress Creep and to Recover Original Cross-Link
Density after Reprocessing: Quantitative Theory and Experiments. Macromolecules.

[46] Li L., Chen X., Jin K., Rusayyis M. B., Torkelson J. M. (2021). Arresting
Elevated-Temperature Creep and Achieving Full Cross-Link Density Recovery
in Reprocessable Polymer Networks and Network Composites via Nitroxide-Mediated
Dynamic Chemistry. Macromolecules.

[47] Khonakdar H. A., Morshedian J., Wagenknecht U., Jafari S. H. (2003). An Investigation
of Chemical Crosslinking Effect on Properties of High-Density Polyethylene. Polymer (Guildf).

[48] Fenimore L. M., Bin Rusayyis M. A., Onsager C. C., Grayson M. A., Torkelson J. M. (2024). Reprocessable
Polymer Networks Containing Sulfur-Based, Percolated Dynamic Covalent
Cross-Links and Percolated or Non-Percolated, Static Cross-Links. Macromol. Rapid Commun..

[49] Wang L., Wan D., Qiu J., Tang T. (2012). Effects of Long Chain Branches on
the Crystallization and Foaming Behaviors of Polypropylene-g-Poly­(Ethylene-Co-1-Butene)
Graft Copolymers with Well-Defined Molecular Structures. Polymer (Guildf).

[50] Wang L., Jiang Z. W., Liu F., Zhang Z. J., Tang T. (2014). Effects of
Branches on the Crystallization Kinetics of Polypropylene-g- Polystyrene
and Polypropylene-g-Poly­(n-Butyl Acrylate) Graft Copolymers with Well-Defined
Molecular Structures. Chinese Journal of Polymer
Science (English Edition).

[51] Jeziorny A. (1978). Parameters
Characterizing the Kinetics of the Non-Isothermal Crystallization
of Poly­(Ethylene Terephthalate) Determined by d.s.c. Polymer (Guildf).

[52] Kissinger H. E. (1956). Variation
of Peak Temperature With Heating Rate In Differential Thermal Analysis. J. Res. Natl. Bur. Stand..

[53] Sadri M., Barbour A., Thornell T. L., Newman J. K., Qiang Z. (2025). Composition–Structure–Property
Relationships of Polyethylene Vitrimers Crosslinked by 8-Arm Polyhedral
Oligomeric Silsesquioxane. Soft Matter.

[54] Kolesov I.
S., Androsch R., Radusch H. J. (2005). Effect of Crystal Morphology and
Crystallinity on the Mechanical α-and β-Relaxation Processes
of Short-Chain Branched Polyethylene. Macromolecules.

[55] Pick L. T., Harkin-Jones E. (2005). An Investigation
of the Relationship between Thermal
Relaxations and the Impact Performance of Rotationally Moulded Linear
Low Density Polyethylenes. In Proceedings of
the Institution of Mechanical Engineers, Part L: Journal of Materials:
Design and Applications.

[56] Sharif A., Mohammadi N., Ghaffarian S. R. (2009). Model Prediction of the ESCE of Semicrystalline
Polyethylene: Effects of Melt Cooling Rate. J. Appl. Polym. Sci..

[57] Di
Lorenzo M. L., Silvestre C. (1999). Non-Isothermal Crystallization of
Polymers. Prog. Polym. Sci..

[58] Yang B., Yang M., Wang W. J., Zhu S. (2012). Effect of Long Chain
Branching on Nonisothermal Crystallization Behavior of Polyethylenes
Synthesized with Constrained Geometry Catalyst. Polym. Eng. Sci..

[59] Avrami M. (1939). Kinetics of
Phase Change. I: General Theory. J. Chem. Phys..

[60] Avrami M. (1941). Granulation,
Phase Change, and Microstructure Kinetics of Phase Change. III. J. Chem. Phys..

[61] Gupta S., Yuan X., Chung T. C. M., Cakmak M., Weiss R. A. (2014). Isothermal
and Non-Isothermal Crystallization Kinetics of Hydroxyl-Functionalized
Polypropylene. Polymer (Guildf).

[62] Kamal M. R., Chu E. (1983). Isothermal and Nonisothermal
Crystallization of Polyethylene. Polym. Eng.
Sci..

[63] Suh J., White J. L. (2007). Comparative Study on Quiescent Crystallization Kinetics
of Isotactic Polyolefins. J. Appl. Polym. Sci..

[64] Cupta A. K., Rana S. K., Deopura B. (1994). 1. Crystallization
Kinetics of High-Density
Polyethylene/ Linear Low-Density Polyethylene Blend. J. Appl. Polym. Sci..

[65] He C., Cao X., Huo G., Luo S., He X. (2015). Non-Isothermal Crystallization
Behaviour and Kinetics of LLDPE/REDMUD Blends. Polymers & Polymer Composites.

[66] Liu Y., Yang Q., Li G. (2008). Nonisothermal
Crystallization Behavior
of LLDPE/Glass Fiber Composite. J. Appl. Polym.
Sci..

[67] Cebe P. (1988). Non-Isothermal
Crystallization of Poly­(Etheretherket0ne) Aromatic Polymer Composite. Polym. Compos.

[68] Herrero C. R., Acosta J. L. (1994). Effect of Poly­(Epichlorhydrin) on the Crystallization
and Compatibility Behavior of Poly­(Ethylene Oxide)/Polyphosphazene
Blends. Polym. J..

[69] Albano C., Papa J., Ichazo M., González J., Ustariz C. (2003). Application of Different Macrokinetic
Models to the
Isothermal Crystallization of PP/Talc Blends. Compos Struct.

