# Supplementary material for: Understanding Non-isothermal Crystallization Behaviors of Polyethylene-Derived Vitrimers
Source: Macromolecules. 2025 Aug 20;58(17):9348–57. doi: 10.1021/acs.macromol.5c01725 (PMC12424294; doi:10.1021/acs.macromol.5c01725)
Supplement: Supplementary file 1 [file ma5c01725_si_001.pdf]

Supporting Information for:

Understanding non-isothermal crystallization behaviors of polyethylene-derived vitrimers

*Sara Valdez and Zhe Qiang**

School of Polymer Science and Engineering, The University of Southern Mississippi, Hattiesburg, MS 39406

Corresponding author: Zhe Qiang Email: zhe.qiang@usm.edu

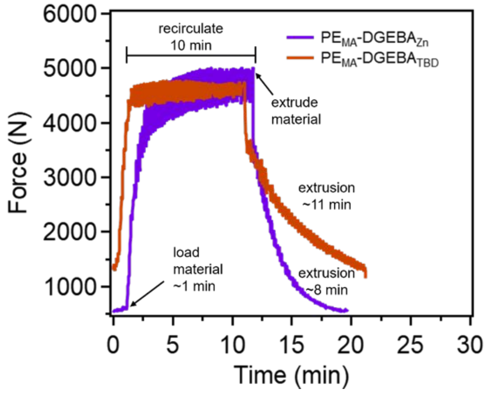

Figure S1. Force output from the microcompounder for typical vitrimer samples, highlighting the plateau of the material's viscosity.

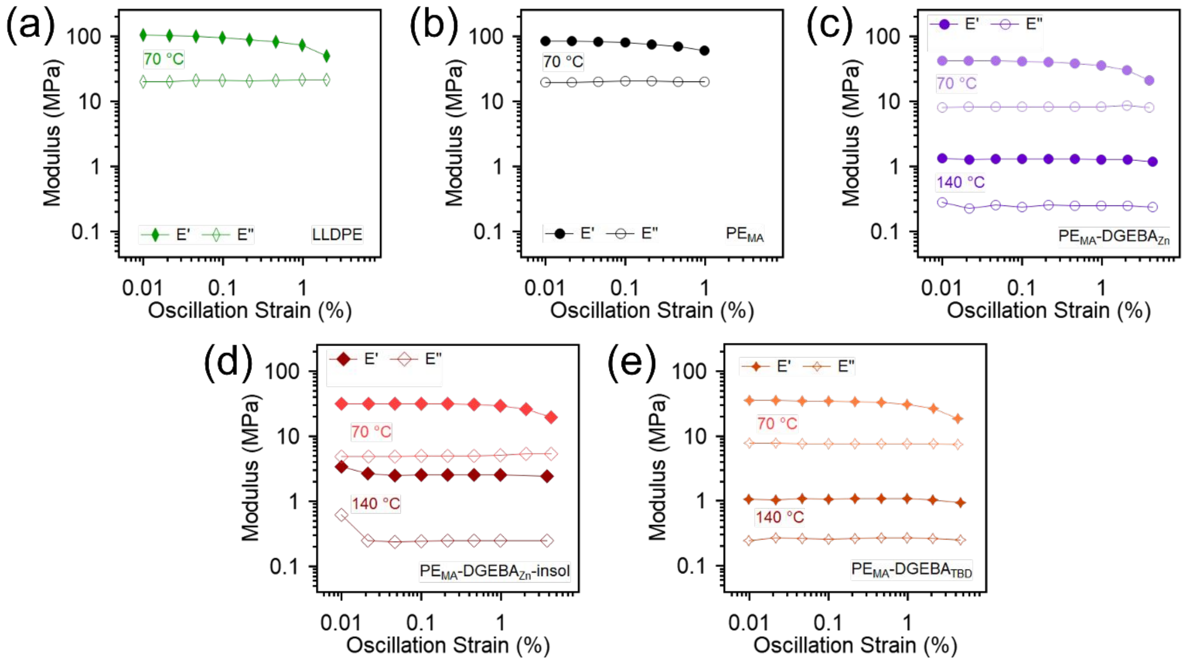

Figure S2. Modulus-oscillatory strain plots at 70 °C to highlight LVR for (a) LLDPE and (b) PE_{MA} and at 70 and 140 °C for (c) PE_{MA}-$DGEBA_{Zn}$, (d) PE_{MA}-$DGEBA_{Zn}$-insol, and (e) PE_{MA}-$DGEBA_{TBD}$.

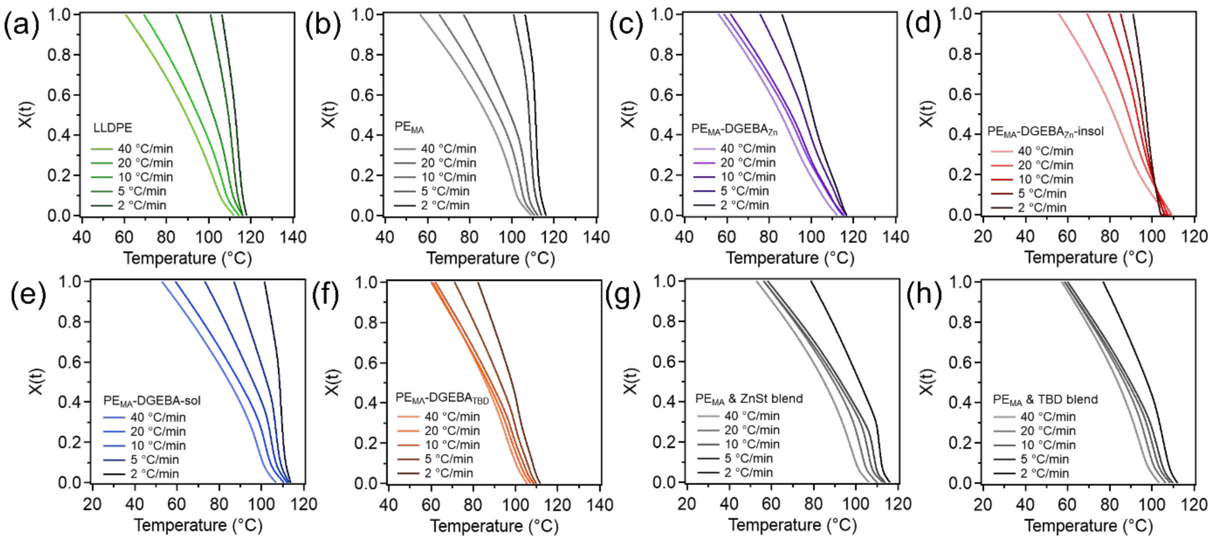

Figure S3. Development of the relative crystallinity while cooling for non-isothermal crystallization for (a) LLDPE, (b) PE_{MA}, (c) PE_{MA}-$DGEBA_{Zn}$, (d) PE_{MA}-$DGEBA_{Zn}$-insol, (e) PE_{MA}-DGEBA-sol, and (f) PE_{MA}-$DGEBA_{TBD}$, (g) PE_{MA} & ZnSt blend, and (h) PE_{MA} & TBD blend.

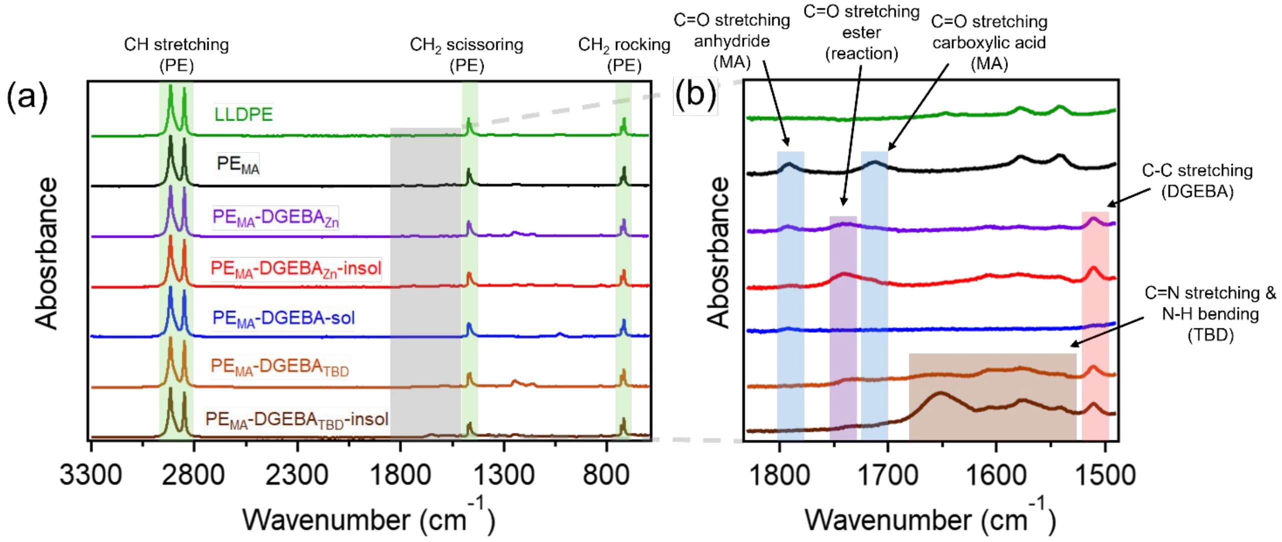

Figure S4. (a) FTIR spectra and (b) closeup of the range highlighting the esterification reaction of all the materials.

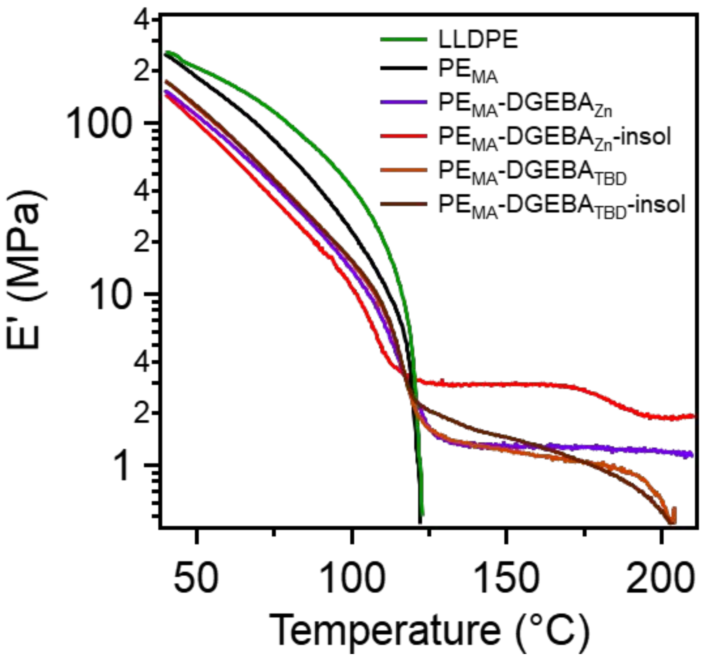

Figure S5. Storage modulus-temperature plot for the materials.

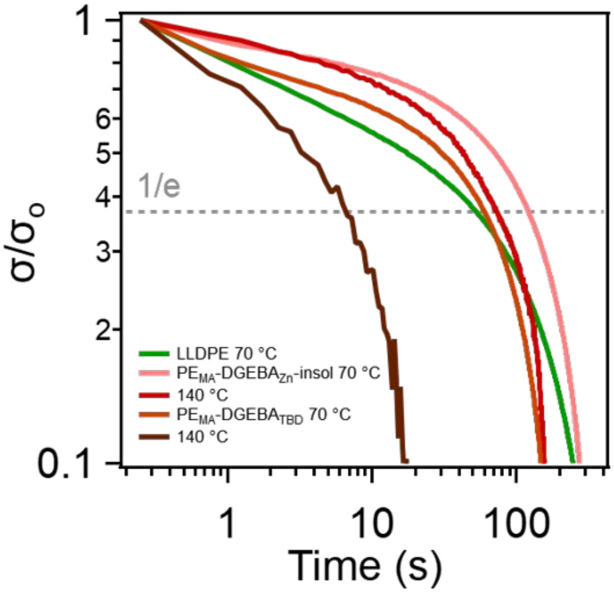

Figure S6. Stress relaxation plot for the materials all with a strain of 0.2%.

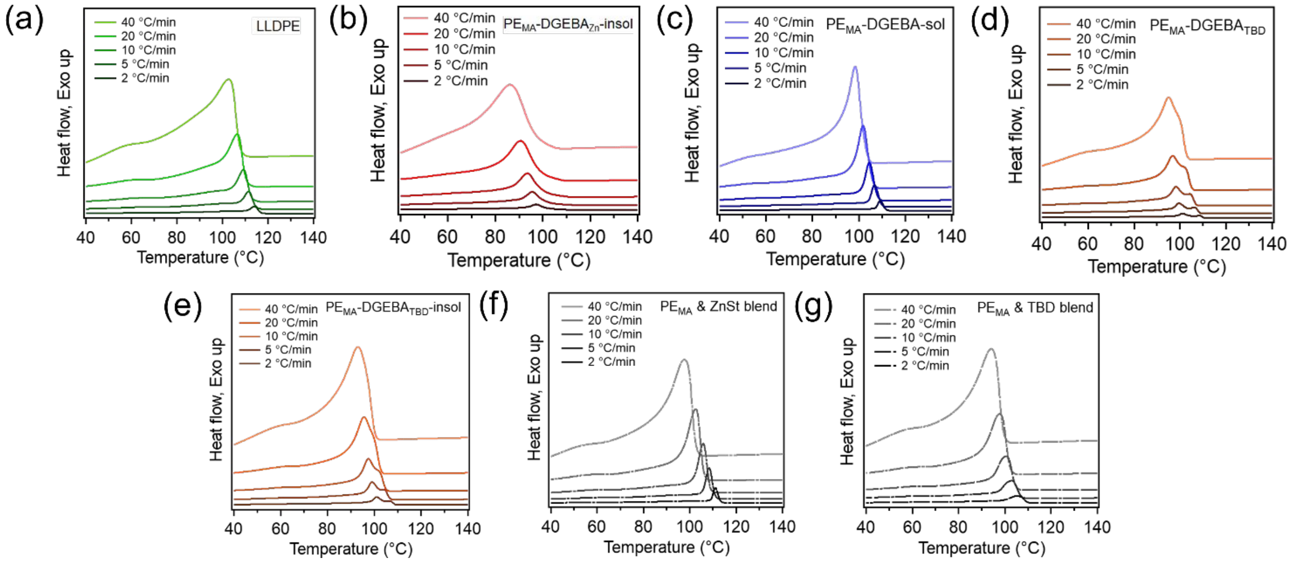

Figure S7. DSC thermograms with various cooling rates for (a) LLDPE, (b) PE$_{MA}$-DGEBA$_{Zn}$-insol, (c) PE$_{MA}$-DGEBA-sol, (c) PE$_{MA}$-DGEBA$_{TBD}$, (e) PE$_{MA}$-DGEBA$_{TBD}$-insol, (f) PE$_{MA}$ & ZnSt blend, and (g) PE$_{MA}$ & TBD blend.

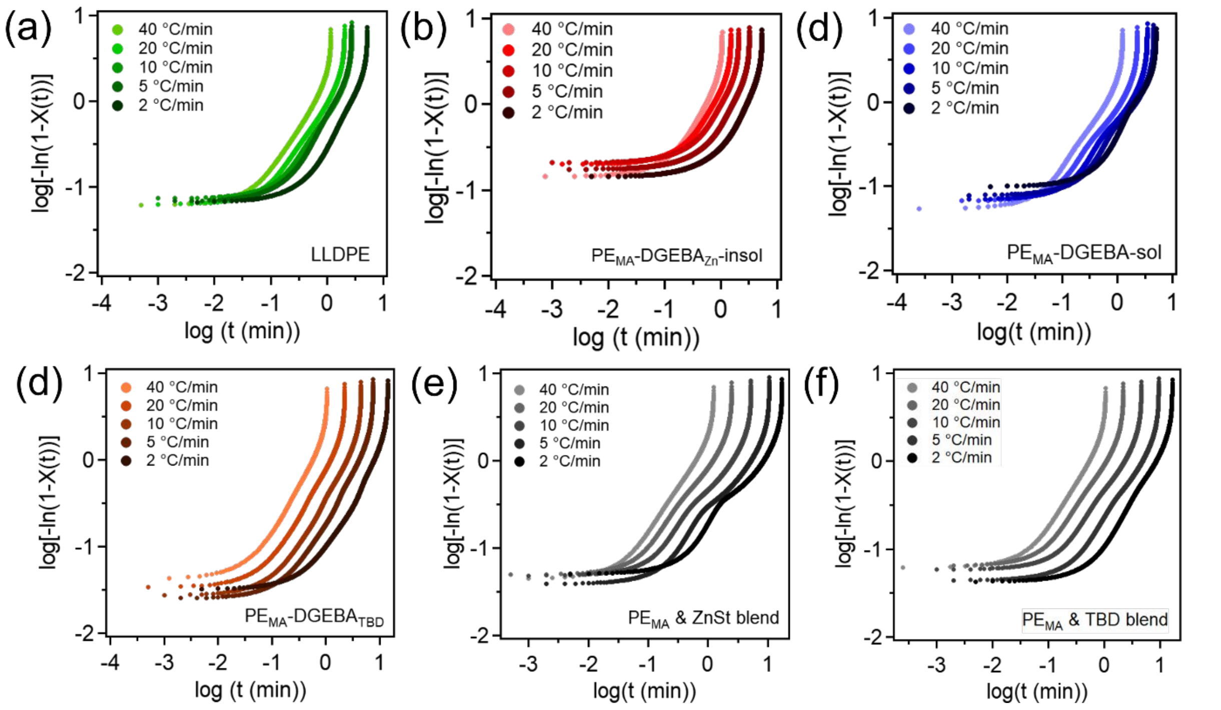

Figure S2. Avrami plots for (a) LLDPE, (b) PE$_{MA}$-DGEBA$_{Zn}$-insol, (c) PE$_{MA}$-DGEBA-sol, and (d) PE$_{MA}$-DGEBA$_{TBD}$, (e) PE$_{MA}$ & ZnSt blend, and (f) PE$_{MA}$ & TBD blend.

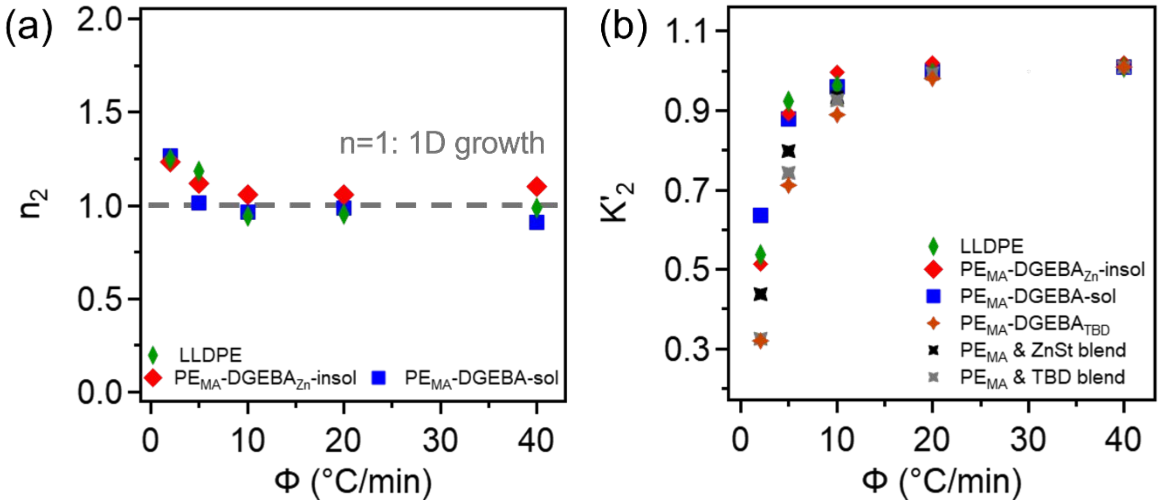

Figure S3. (a) Avrami exponents and (b) Jeziorny-modified Avrami constant for the materials from the second region of their corresponding Avrami plots.

Table S1. Table listing T_{onset}, Tp, $t_{1/2}$, n_2, K'_2, and $\chi_{c,\,PE}$ for all the samples.

Sample	ϕ (°C/min)	T_{onset} (°C)	T_p (°C)	$t_{1/2}$ (min)	n_2	K'_2	$\chi_{c,\,PE}$ (%)
LLDPE	2	116.64	114.07	2.7	1.25	0.537	38.4
	5	114.42	111.57	1.4	1.19	0.924	37.9
	10	112.51	109.27	1.4	0.944	0.962	37.7
	20	110.25	106.52	0.97	0.954	0.995	37.5
	40	107.50	102.87	0.57	0.989	1.01	-
PE_{MA}	2	113.28	111.35	2.5	1.37	0.795	41.3
	5	111.06	108.88	1.6	1.11	0.953	40.5
	10	109.05	106.63	1.3	1.04	0.990	40.0
	20	106.68	103.95	0.86	0.944	1.01	39.6
	40	103.80	100.70	0.53	0.823	1.01	-

PE_{MA}-$DGEBA_{Zn}$	2	115.96	99.49/113.76	8.2	0.830	0.276	33.1
	5	114.59	97.71/111.84	4.0	0.791	0.693	32.8
	10	113.17	96.58/110.09	2.4	0.817	0.855	32.7
	20	111.38	94.57/107.05	1.3	0.799	0.960	32.5
	40	109.06	91.23/104.13	0.67	0.844	0.992	-
PE_{MA}-$DGEBA_{Zn}$-insol	2	101.61	97.21	3.4	1.23	0.514	30.9
	5	101.07	95.45	1.8	1.12	0.893	30.6
	10	100.07	93.42	1.1	1.06	0.998	30.1
	20	99.26	90.71	0.77	1.06	1.02	29.6
	40	97.70	86.15	0.56	1.10	1.02	-
PE_{MA}-DGEBA-sol	2	111.49	109.19	2.6	1.27	0.637	34.1
	5	109.63	106.84	2.1	1.02	0.879	33.8
	10	107.96	104.53	1.6	0.964	0.961	33.7
	20	105.73	101.83	1.1	0.985	1.00	33.6
	40	102.84	98.43	0.55	0.909	1.01	-
PE_{MA}-$DGEBA_{TBD}$	2	110.25	100.93/108.27	6.7	0.829	0.320	34.7
	5	108.42	99.47/106.1	3.4	0.897	0.711	34.0
	10	106.88	98.2/104.07	2.0	0.922	0.889	33.5
	20	105.05	96.89/102.1	1.0	0.990	0.981	33.0
	40	102.89	95.05/99.81	0.49	1.04	1.01	-
PE_{MA}-$DGEBA_{TBD}$-insol	2	-	100.84	-	-	-	-
	5	-	98.97	-	-	-	-
	10	-	97.48	-	-	-	-
	20	-	95.62	-	-	-	-
	40	-	93.02	-	-	-	-
PE_{MA} & ZnSt blend	2	113.15	111.04	7.2	1.10	0.438	36.6
	5	110.76	108.32	4.2	1.05	0.799	36.1
	10	108.58	105.82	2.1	0.900	0.935	35.7
	20	105.94	102.50	1.0	0.929	0.994	35.2

	40	102.52	97.71	0.53	0.978	1.01	-
PE_{MA} & TBD blend	2	109.60	105.45	7.1	1.07	0.325	35.6
	5	106.73	103.09	4.0	0.947	0.743	34.4
	10	104.43	100.68	2.0	0.909	0.927	33.7
	20	102.02	97.77	0.98	0.918	0.994	33.4
	40	99.19	94.14	0.49	0.929	1.01	-

Table S2. Table listing the relaxation times when σ/σ_o=1/e (τ) for all the materials.

Sample	τ (s)
LLDPE 70 °C	52.3
PE_{MA} 70 °C	48.3
PE_{MA}-$DGEBA_{Zn}$ 70 °C	101
PE_{MA}-$DGEBA_{Zn}$ 140 °C	83.3
PE_{MA}-$DGEBA_{Zn}$-insol 70 °C	121
PE_{MA}-$DGEBA_{Zn}$-insol 140 °C	72.0
PE_{MA}-$DGEBA_{TBD}$ 70 °C	58.0
PE_{MA}-$DGEBA_{TBD}$ 140 °C	6.50
